# Predictors of high healthcare costs in elderly patients with liver cancer in end-of-life: a longitudinal population-based study

**DOI:** 10.1186/s12885-017-3561-5

**Published:** 2017-08-24

**Authors:** Jui-Kun Chiang, Yee-Hsin Kao

**Affiliations:** 1 Department of Family Medicine, Buddhist Dalin Tzu Chi Hospital, Chiayi, Taiwan; 2 Department of Family Medicine, Tainan Municipal Hospital, 670 Chung-Te Road, Tainan, 701 Taiwan

**Keywords:** Healthcare costs, Elderly, Liver cancer, Last month of life (LML), End of life (EOL)

## Abstract

**Background:**

Studies have indicated a pervasive pattern of decreasing healthcare costs during elderly patients’ last year of life. The aim of this study was to explore the predictors of high healthcare costs (HC) in elderly liver cancer patients in Taiwan during their last month of life (LML).

**Methods:**

Costs of hospitalization, outpatient visits, aggressiveness of care, and associated costs for elderly (age ≥ 65 y) patients with liver cancer in the LML were analyzed using a national insurance database. An HC was defined as being greater than the 90th percentile (US $5093) in the LML, amounting to 38.95% of total healthcare costs.

**Results:**

We enrolled 2121 subjects who died during 1997–2011. Mean healthcare costs per person in their LML were US $8042 ± 3477 in the HC group and US $1407 ± 1464 in the non-HC group (*p* < 0.001). For patients receiving aggressive end-of-life (EOL) cancer care (e.g. intensive care, cardiopulmonary resuscitation, anticancer treatment, and a high number of admission days), comorbidities of chronic kidney disease, esophageal bleeding, and receiving opioids in the LML, were significantly independent positive predictors of HCs; but admission times, comorbidities of ascites, and hypertension were negative predictors.

**Conclusion:**

These findings could inform healthcare providers by avoiding aggressive treatments during EOL for elderly patients with liver cancer and to save on healthcare costs. Shorter admission days and more admission times in the last month of life could decrease healthcare costs.

**Electronic supplementary material:**

The online version of this article (doi:10.1186/s12885-017-3561-5) contains supplementary material, which is available to authorized users.

## Background

Liver cancer is the third most common cause of cancer death worldwide and has a high fatality rate (overall ratio of mortality to incidence of 0.93) [1]. It was one of the leading causes of cancer death in Taiwan (18.3% in 2013) [2]. Elderly people have an 11-fold higher incidence of cancer compared with those younger than 65 years [3]. In Taiwan, liver cancer accounts for 24.9% of people among all cancer death in the elderly population [4].

Studies have indicated a pervasive pattern of decreasing healthcare costs during elderly patients’ last year of life [5–7]. Elderly patients with cancer receive fewer aggressive treatments and resource-intensive care at EOL because they receive less chemotherapy [8–11] and fewer life-extending treatments, including intensive care unit (ICU) care [8, 9, 12–15], CPR [8, 16], intubation, and mechanical ventilator support [8, 17–19]. The quality of EOL care is a crucial indicator of the quality of cancer care. Certain quality indicators for EOL cancer care have been proposed and validated in the United States [20, 21] and Canada [22, 23] according to the following: chemotherapy within 2 weeks of death, more than one emergency room (ER) visit in the last month of life (LML), more than one hospitalization in the LML, at least one admission to an ICU in the LML, or death in hospital. Hospice care, however, aims to relieve symptoms, pain, and suffering during EOL and provides a multitude of benefits including prolonged survival and improved quality of life [24, 25].

The direct healthcare costs of treating cancers have increased dramatically worldwide [26]. They have also risen in Taiwan, where hospitalization costs have increased 172% from 1999 (US $3,227,790) to 2007 (US $5,524,095) [27]. These increases have lead to financial hardship for some patients and a greater burden on Taiwan’s health insurance system.

Interest in EOL healthcare costs for patients with cancer has increased, and many studies on these costs have based their findings on administrative health data [28]. Yabroff et al. reported that the mean costs of cancer care are highest in the initial period after cancer diagnosis and in last year of life [29]. In the United States, 25% of healthcare costs are devoted to EOL care for elderly patients, particularly in their LML [30, 31]. Most of these costs result from life-sustaining care and a high number of admission days in the LML [32]. One review article reported a reduction in last-month costs for elderly patients with cancer, attributing the reduction to decreased use of hospital services or increased use of palliative care [28]. However little is known about the factors associated with high healthcare costs (HCs) for elderly patients with liver cancer in their LML.

According to the Ministry of the Interior’s statistics on life expectancy, Taiwan’s population moved from ‘aging’ to ‘aged’, and the percentage of elderly was 11.75% in 2014 [33]. However, the percentage of mortality for elderly patients was also increased, and reached 59.6% in 2011. Liver cancer was the leading cause of cancer death in Taiwan, and the percentage for elderly patients with liver cancer in all cancer death was 11.0% in 2011 [2]. Using Taiwan’s National Health Insurance (NHI) database, this study evaluated the factors associated with HCs for elderly patients with liver cancer during their LML.

## Methods

### Data source

In this national population-based cohort study, data obtained from Taiwan’s NHI Research Database (NHIRD) were analyzed. Implemented in March 1995, Taiwan’s NHI program is a single-payer health insurance system that covered approximately 99.9% of the total population of Taiwan in 2012 [34]. In Taiwan, patients with cancer must be examined to receive a catastrophic illness certificate (CIC). We used Taiwan’s 2000 Longitudinal Health Insurance Database (LHID2000), a subset of the NHIRD containing all the original claims data from 1,000,000 individuals randomly sampled from the registry of the NHIRD in 2000. Patients were linked to the LHID2000 to obtain the hospital care and outpatient data collected from 1996 to 2011.

### Identification

All elderly patients who received a first-time diagnosis of liver cancer between January 1, 1997 and December 31, 2011 were included in our study. The International Classification of Diseases, Ninth Revision, Clinical Modification (ICD-9-CM) and A codes were used to define liver cancer (155, 155.0, 155.1, A095). The NHIRD and catastrophic illness database were used to identify patients with liver cancer. Patients younger than 65 years were excluded. The variables selected for analyses in this study included demographic characteristics, comorbidities, opioids use, cares, treatments, and medical expenditures in the last month of life. The basic demographic characteristics, such as gender, birthday and socioeconomic status, were attained from the basic files of the claim data. The diagnostic codes for comorbidities for elderly patients with liver cancer were as below. The ICD-9-CM and A codes were used to define liver cirrhosis (571.5, 571.6, and 571.2), hepatitis B virus (HBV) (070.20–070.23, 070.30–070.33, V0261, and A046), hepatitis C virus (HCV) (070.41, 070.44, 070.51, 070.54, V0262, and A046), esophageal varices with bleeding (456.0 and 456.20), ascites (789.5), stroke (430–437), chronic kidney disease (CKD) (585), and hemodialysis (V451). To increase validity, those who had at least 3 diagnoses of diabetes (ICD-9-CM: 250 and A code A181) and at least 3 diagnoses of hypertension (ICD-9-CM: 401–405 and A code A26) for either outpatient or inpatient care within 365 calendar days were considered to have a diagnosis of diabetes or hypertension [35, 36]. The nationwide prevalence rate of hypertension was approximately 47% for elderly patients in Taiwan [37]. The cares and treatments during the last month of life for elderly patients with liver cancer included ICU admission, intubation, mechanical ventilation, admission days, admission times, emergency room visit, receiving anti-cancer therapy, and hospice care. There were special codes and payments for the above therapies to be identified from the claim data.

### Definition of variables

#### Healthcare costs

The healthcare costs of the inpatient and outpatient charges were summed during the last month as well as the last second and third month before death. Regarding US dollars (US $) and New Taiwan dollars (NT $), the exchange rates in 2006 were US $1.00 = NT $32.53 [27].

#### HC and non-HC groups

According to healthcare costs in the LML, patients were divided into the high healthcare cost (HC) group, which included those with costs greater than the 90th percentile (US $5093), amounting to 38.95% of total healthcare costs, and non-HC group, which included those with costs lower than the 90th percentile.

#### Charlson Comorbidity index

The Charlson comorbidity index (CCI) was calculated by examining ICD-9-CM diagnosis and procedure codes recorded in the year prior to diagnosis according to the Deyo method and applied to inpatient and outpatients claims, as in Klabundle et al. [38–40].

#### Opioid use

Opioid use was defined as patients’ receiving any opioid medicines including oral forms, injection forms, or fentanyl transdermal patches.

The protocol for this study was reviewed and approved by the Research Ethics Committee of the Buddhist Dalin Tzu Chi Hospital, Taiwan (No. B10301001). Because the NHIRD files only contain deidentified secondary data, the review board waived the need for informed consent.

### Statistical analysis

The continuous variables were expressed as mean ± standard deviation (SD), and categorical variables were presented by frequency and percentage. In univariate analysis, a 2-sample *t* test, Wilcoxon rank-sum test, chi-squared test, and Fisher’s exact test were used to examine the differences in the distributions of continuous or categorical variables between the 2 groups. Survival probability estimates after cancer diagnosis were analyzed using the Kaplan–Meier method. HC was defined as being greater than the 90th percentile (US $5093) for patients in their LML. Multiple logistic regressions with the stepwise variable selection procedure were performed to identify crucial predictors of high healthcare cost. Generalized additive models were fitted to detect potential nonlinear effects of continuous covariates [41].

The goodness-of-fit (GOF) of the final logistic regression model was assessed by applying the estimated area under the receiver operating characteristic (ROC) curve and Hosmer–Lemeshow GOF test. Regarding the Hosmer–Lemeshow test, *p* > 0.05 indicated GOF. Finally, regression diagnostics for residual analysis, detection of influential cases, and checks of multicollinearity were used to discover any problems with the model or data. All statistical analyses were performed using the R 3.0.2 software (R Foundation for Statistical Computing, Vienna, Austria). Two-sided *p* ≤ 0.05 was considered significant.

## Results

**Fig. 1:**
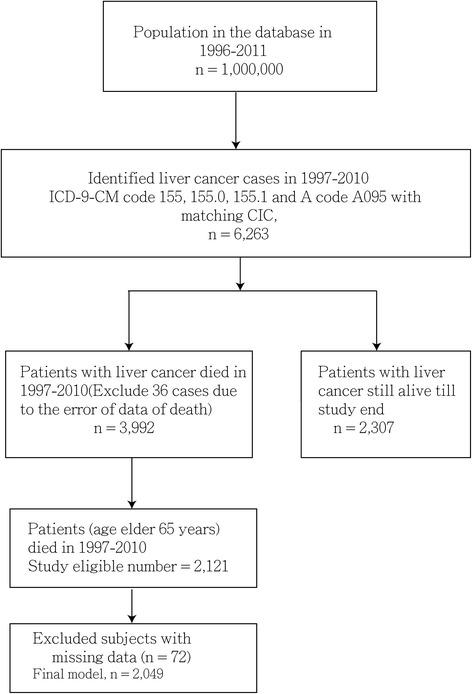
Study flow chart. Abbreviations: ICD-9-CM, International Classification of Diseases, Ninth Revision, Clinical Modification; CIC, catastrophic illness certificate

**Fig. 2:**
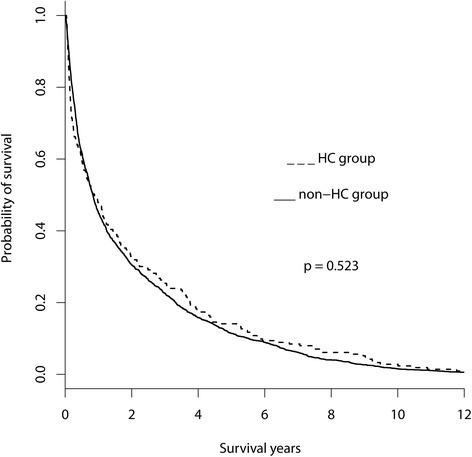
The survival curves for the high healthcare costs (HC) and non-HC groups

A total of 2121 elderly (age ≥ 65 y) patients were identified. The study flow chart is shown in Fig. 1. The variables for analyses in this study were listed in Tables 1 and 2 included gender, age, survival years, comorbidities (including complications of cirrhosis), opioids use, urbanization level, admission days and times, and end-of-life cancer care (including ICU admission, receiving anti-cancer therapy, intubation, mechanical ventilation, ER visits, admission times and admission days in the last month of their life). Regarding the comorbidities and their intensity, including CCI, hypertension, HBV or HCV infection, and cirrhosis, no significant difference was observed between the HC and non-HC groups. However, the HC group had more comorbidities such as chronic kidney disease (CKD) (26 (12.2%) vs 129 (6.8%), *p* = .008), hemodialysis (33 (17.8%) vs 62 (3.2%), *p* < 0.001), esophageal varices with bleeding (38 (15.5%) vs 149 (7.8%), *p* < 0.001), but less portion of ascites (40 (18.8%) vs 479 (25.1%), *p* = 0.044) than those of the non-HC group. Regarding other characteristics, the HC group had higher proportions of patients with opioid medicine use (66 (31.0%) vs 393 (20.6%), *p* = 0.001) (Table 1). No significant difference in survival probability after cancer diagnosis was found between the 2 groups (*p* = 0.523) (Fig. 2). The median survival in years from cancer diagnosis to death for the HC and non-HC groups was 0.88 and 0.84, respectively. The mean ± SD (median) days from hospice enrollment to death were 41.48 ± 92.94 (16.00). In this study, urbanization levels were divided into 3 strata: urban, suburban, and rural. No significant difference between the HC and non-HC groups was found regarding these urbanization levels.

**Table 1:** Demographic characteristics of elderly patients with liver cancer

Characteristics	Total	Non-HC group	HC group	*p* value
Number of patients, *n* (%)	2121	1909(90%)	212(10%)	
Gender				0.133
Male	1349(63.6%)	1224(64.2%)	125(58.7%)	
Female	772(36.4%)	684(35.8%)	88(41.3%)	
Age on death, years	75.8 ± 6.5	75.3 ± 6.6	74.7 ± 6.4	0.233
Survival after cancer diagnosis, years [mean (median)]	1.94(0.80)	1.92(0.84)	2.10(0.88)	0.523
Diabetes	296(14.0%)	268(10.4%)	28(13.1%)	0.835
Hypertension	426(20.1%)	383(20.1%)	43(20.2%)	1
Stroke	164(7.7%)	144(7.5%)	20(9.4%)	0.343
HBV	436(20.6%)	384(20.1%)	52(24.4%)	0.152
HCV	621(29.3%)	556(29.1%)	65(30.5%)	0.692
Liver cirrhosis	1275(60.1%)	1139(59.7%)	136(63.8%)	0.268
EVB	18.7(8.8%)	149(7.8%)	38(17.8%)	<0.001
Ascites	519(24.5%)	479(25.1%)	40(18.8%)	0.044
CKD	155(7.3%)	129(6.8%)	26(12.2%)	0.008
Hemodialysis	95(4.5%)	62(3.2%)	33(15.5%)	<0.001
CCI (scores)	3.64 ± 2.36	3.62 ± 2.32	3.80 ± 2.65	0.534
Opioids use	459(21.6%)	393(20.6%)	66(31.0%)	0.001
Urbanization level				
Urban	949(44.7%)	842(44.1%)	107(50.2%)	0.095
Suburban	775(36.5%)	702(36.8%)	73(34.3%)	0.500
Rural	397(18.7%)	364(19.1%)	33(15.5%)	0.229

Abbreviations: *HC group* high healthcare cost group (defined as those whose costs in the LML were greater than the 90th percentile (US $5093); non-HC group, non-high healthcare cost group (defined as those whose costs in the LML were lower than the 90th percentile), *HBV* hepatitis B virus, *HCV* hepatitis C virus, *EVB* esophageal varices with bleeding, *CKD* chronic kidney disease, *CCI* Charlson comorbidity index

**Table 2:** Comparison of the aggressiveness of EOL care and cost between the HC and non-HC groups in the LML

Variables	Total	Non-HC group	HC group	*p* value
Number, *n* (%)	2121	1909(90%)	212(10%)	
ICU admission	308(14.5%)	174(9.1%)	134(62.9%)	<0.001
Intubation	252(11.9%)	168(8.8%)	84(39.4%)	<0.001
Mechanical ventilation	308(14.5%)	194(10.2%)	114(53.5%)	<0.001
Admission days	10.7 ± 10.9	9.1 ± 10.1	25.0 ± 6.1	<0.001
Admission times	0.8 ± 0.7	0.8 ± 0.8	1.2 ± 0.4	<0.001
ER visit (times)	0.7 ± 1.1	0.7 ± 1.1	0.7 ± 0.9	0.694
Receiving anti-cancer therapy as below	155(7.3%)	108(5.7%)	47(22.1%)	<0.001
TACE	75(3.5%)	56(2.9%)	19(8.9%)	<0.001
Radiotherapy	57(2.7%)	34(1.8%)	23(10.8%)	<0.001
Chemotherapy	15(0.7%)	14(0.7%)	1(0.5%)	1
HAIC	14(0.7%)	11(0.6%)	3(1.4%)	0.159
PEI	5(0.2%)	3(0.2%)	2(0.9%)	0.082
RFA	7(0.3%)	6(0.3%)	1(0.5%)	0.524
Hospice care (yes)	405(19.1%)	380(19.9%)	25(11.7%)	0.003
Death in a hospital	821(38.7%)	679(35.6%)	142(66.7%)	<0.001
Cost (US dollars)	2073 ± 2667	1407 ± 1464	8042 ± 3477	<0.001

Abbreviations: *ICU* intensive care unit, *ER* emergency room, *TACE* transcatheter arterial chemoembolization, *HAIC* hepatic artery infusion chemotherapy, *PEI* percutaneous ethanol injection, *RFA* radiofrequency ablation

The aggressiveness of EOL care in the HC and non-HC group was compared by univariate analysis (Table 2). Significantly larger proportions of the HC group had received ICU treatment (134 (62.9%) vs 174 (9.1%), *p* < 0.001), use of intubation (84 (39.4%) vs 168 (8.8%), *p* < .001), or use of mechanical ventilation (114 (53.5%) vs 194 (10.2%), *p* < 0.001), had a greater number of admission days (25.0 vs 9.1, *p* < 0.001) or times of admission (1.2 vs 0.8, *p* < 0.001), had received anticancer treatment (47 (22.1%) vs 108 (5.7%), *p* < 0.001), and died in a hospital (142 (66.7%) vs 679 (35.6%), *p* < 0.001). The mean healthcare costs in the HC group during the LML were higher than those in the non-HC group (US $8042 ± 3477 vs US $1407 ± 1464, *p* < .001).

The mean total healthcare costs in the third month before death were US $721; the costs in the second month before death were US $897, and the costs in the last month before death were US $2073. The healthcare costs for elderly patients with liver cancer in the LML comprised inpatient costs of US $2056 (99.2%) and outpatient costs of US $17 (0.8%). A total of 212 patients (10%) in the HC group accounted for 38.95% of the total cost in the LML (US $1,712,608 of US $4,396,456). Predictors of HCs were examined using multiple logistic regression analysis. We found that for patients receiving aggressive end-of-life (EOL) cancer care, such as ICU care (odds ratio (OR): 13.34, 95% confidence interval (CI): 7.69–23.14, *p* < 0.001), ventilator support (OR: 3.46, 95% CI: 2.04–5.87, *p* < 0.001), anticancer treatment (OR: 2.72, 95% CI: 1.60–4.62, *p* < 0.001), and a higher number of admission days (OR: 1.25, 95% CI: 1.21–1.30, *p* < 0.001), comorbidities such as CKD (OR: 2.92, 95% CI: 1.44–5.93, *p* = 0.003), esophageal bleeding (OR: 2.26, 95% CI: 1.17–4.36, *p* = 0.015), and receiving opioid medicine (OR: 1.85, 95% CI: 1.17–2.91, *p* = 0.009) in the LML were significant independent positive predictors for HCs. However, patients with more admission times (OR: 0.43, 95% CI: 0.27–0.68, *p* < 0.001), comorbidities of ascites (OR: 0.34, 95% CI: 0.20–0.59, *p* < 0.001) and hypertension (OR: 0.59, 95% CI: 0.36–0.99, *p* = 0.046) were significantly negative predictors for HCs (Table 3).

**Table 3:** Factors associated with HCs for elderly patients with liver cancer in their LML

Covariates	Estimate	O.R.	95% C.I.	*p* value
ICU	2.59	13.34	7.69–23.14	<0.001
Ventilator	1.24	3.46	2.04–5.87	<0.001
Anti-cancer treatments^a^	1.00	2.72	1.60–4.62	<0.001
Admission days	0.22	1.25	1.21–1.30	<0.001
Admission times	−0.85	0.43	0.27–0.68	<0.001
CKD	1.07	2.92	1.44–5.93	0.003
EVB	0.81	2.26	1.17–4.36	0.015
Opioids use	0.61	1.85	1.17–2.91	0.009
Ascites	−1.08	0.34	0.20–0.59	<0.001
Hypertension	−0.52	0.59	0.36–0.99	0.046
Intercept	−6.92			<0.001

In this final model, 2049 cases (96.61%) were enrolled for analysis after excluding those (*n* = 72, 3.39%) with missing data

Abbreviations: *ICU* intensive care unit, *CKD* chronic kidney disease, *EVB* esophageal varices with bleeding

^a^Anti-cancer treatments included TACE, Radiotherapy, Chemotherapy, HAIC, PEI, and RFA

**Fig. 3:**
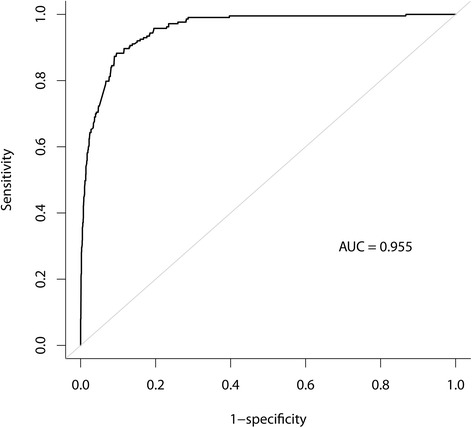
Receiver operating characteristic (ROC) curve for our final fitting model in predicting high healthcare costs (HC) (> 90th percentile, US $5093) in the LML

The Nagelkerke’s R^2^ of this final model was 0.627. Using this model, the area under curve (Fig. 3) were 0.956 (95% CI: 0.943–0.969) (Table 3). The programming code for calculating the probability of HC based on the final model is provided. (Additional file 1: Table S1).

## Discussion

The novel findings of this study are that elderly liver cancer patients with HCs in their LML include patients receiving aggressive EOL cancer care such as ICU care, ventilator support, anticancer treatment, and a high number of admission days. Additionally, comorbidities such as CKD, esophageal varices with bleeding and receiving opioid medicine are significant independent positive predictors of HCs. However, patients with more admission times in the LML, comorbidities of ascites and hypertension were significantly negative predictors of HCs.

The cost of cancer care has increased dramatically in the past 20 years, particularly at EOL [26], thus taxing a burdened health care system. Carlson et al. reported that Medicare patients with healthcare costs greater than the 95th percentile consumed 40% of total Medicare costs from 1998 to 2002 [42]. In the current study, 212 patients (10%) had HCs (> US $5093 per person), accounting for 38.95% of the total healthcare costs of this population. Our slightly lower percentage may be related to differences in health insurance systems.

Elderly patients with advanced liver cancer with HCs received more services including ICU care, mechanical ventilation support, admission days, and anticancer treatments. These results are similar to those of previous studies that have reported that most healthcare costs result from life-sustaining care (e.g. ventilator use and resuscitation) with acute care during the LML [43]. Previous studies have reported that of elderly Taiwanese patients with cancer (all cancer types) from 2001 to 2006, 11.06% received chemotherapy, 18.24% visited the ER multiple times, 10.91% used ICU care, 30.42% received intubation, and 25.39% received mechanical ventilation in their LML [8]. In this study, we found that elderly patients with advanced liver cancer were likely to use ICU care (14.5% vs 10.9%) and were less likely to receive intubation (11.9% vs 30.42%), ventilator support (14.5% vs 25.4%), or chemotherapy (7.3% vs 11.1%) in the LML compared with patients with other types of cancer. Possible explanations for these results are that elderly patients are more likely to have a comorbid disease [44], and the most common causes of liver cancer death are liver-cancer-related or hepatic failure, followed by esophageal varices with bleeding, infections, and renal failure [45], which might incentivize patients to receive intensive care to relieve suffering. Although these patients in this study receiving ICU care and their families were aware of their irreversible conditions, these patients were less likely to receive intubation or mechanical ventilation.

In this study, the percentage of elderly patients with liver cancer who received anticancer treatment in the LML was 7.3%, which was lower than the 11.1% of those who received anticancer treatment among patients with all cancer types in Taiwan and the 20% of those who received anticancer treatment among patients with solid cancer [46]. Furthermore, anticancer treatments drove high EOL costs, which is similar to the findings of a previous study [47]. In this study, we found that transcatheter arterial chemoembolization (TACE) was the most common anticancer treatment for elderly patients with liver cancer in their LML.

Furthermore, we found that the HC group had a larger proportion of opioid use than that of the non-HC group, which drives cancer EOL costs. A possible explanation for the greater use of opioids among patients in the HC group was that patients in this group suffered from increased pain and dyspnea which needed more treatments other than opioids, compared with patients in the non-HC group. However, the quality of life for advanced cancer patients was the aim of hospice care.

Previous studies have found a consistent association between presence of comorbidity and greater resource use [48, 49], which drives healthcare costs in EOL. In this study, we found that elderly patients with advanced liver cancer with ascites were less likely to have HCs in their LML. The explanation for this result might be as below. As listed in Table 1, the proportion of patients with ascites was significantly lower in the HC group (non-HC: 25.1% vs. HC: 18.8%, *p* = 0.044), and then it also showed a significantly negative association with the probability of being in the HC group (OR = 0.34, *p* < 0.001) after adjusting for the effects of the other covariates in Table 3. We speculated that treating the symptom of ascites in terminal HCC patients was less costly and having the symptom of ascites in terminal HCC patients might reduce the willingness to receive more aggressive treatments.

In addition, as listed in Table 2, the mean values of admission days and admission times were significantly higher in the HC group (non-HC: 9.1 days and 0.8 times vs. HC: 25.0 days and 1.2 times, *p* < 0.001 for both), and then admission days still showed a significantly positive association with the probability of being in the HC group (OR = 1.25, *p* < 0.001), but admission times had a significantly negative association with the probability of being in the HC group (OR = 0.43, *p* < 0.001), after adjusting for the effects of the other covariates in Table 3. To investigate the reason(s) behind these two seemly inconsistent results, we dropped admission times from the final logistic regression model of Table 3 and found that the admission days was still significantly positively associated with the probability of being in the HC group (OR = 1.25, *p* < 0.001). In contrast, after we dropped admission days from the final logistic regression model of Table 3, the admission times turns to be positively associated with the probability of being in the HC group (OR = 1.19, *p* = 0.2049) similar to the result seen in Table 2. In summary, admission times had a significantly negative association with the probability of being in the HC group (OR = 0.43, *p* < 0.001), after adjusting for the effects of admission days and the other covariates in Table 3. The interpretation of this result about admission times in Table 3 was that two patients with the same number of admission days in LML, the one with more admission times would tend to be in the non-HC group because they might not have the necessity or opportunity to receive more costly treatments due to shorter stay in each admission.

Based on the results of this study, we constructed a formula for predicting HC risk for elderly liver cancer patients. Health care providers can use the codes provided in the Appendix in addition to available significant factors in the final model as a simple adjunctive method for early prediction of HC. This study had some limitations. One limitation was possible selection bias, which might have occurred because this study was not randomized. Another limitation was our study used administrative data to assess EOL cancer care, which can be an imprecise approach because it depends on the quality of coding and important clinical features regarding patients’ needs and preferences were not collected in administrative data of this analysis [50]. The third limitation was the quality of medical service could not be examined based on the claim data.

## Conclusion

The results of this study provided significant factors including aggressive EOL care, some comorbidities, and opioid use for elderly patients with liver cancer who had HCs in their LML. These findings could inform healthcare providers by avoiding aggressive treatments during EOL for elderly patients with liver cancer and to save on healthcare costs. Shorter admission days and more admission times in LML could decrease healthcare costs.

## Additional file

Additional file 1: Table S1. The codes to calculate the probability of high healthcare cost (HCs) for elderly patients with liver cancer based on our multiple logistic regression model. (DOC 30 kb)

## Abbreviations

CCI: Charlson comorbidity index
CIC: Catastrophic illness certificate
ER: Emergency room
EVB1: Esophageal varices with bleeding
HAIC: Hepatic artery infusion chemotherapy
HBV: Hepatitis B virus
HCV: Hepatitis C virus
ICU: Intensive care unit
NHI: National Health Insurance
NHIRD: National Health Insurance Research Database
PEI: Percutaneous ethanol injection
RFA: Radiofrequency ablation
TACE: Transcatheter arterial chemoembolization

## References

[1] Ferlay J, Shin HR, Bray F, Mathers C, Parkin DM (2010). Estimates of worldwide burden of cancer in 2008: GLOBOCAN 2008. Int J Cancer.

[2] Department of Statistics, Ministry of Health and Welfare. http://www.mohw.gov.tw/cht/DOS/Statistic.aspx?f_list_no=312&fod_list_no=5012. Accessed 19 May 2014.

[3] Lichtman SM (2003). Guidelines for the treatment of elderly cancer patients. Cancer Control.

[4] Ministry of Health and Welfare. Statistics of Causes of Death, Volume 1. http://www.mohw.gov.tw/cht/DOS/Statistic.aspx?f_list_no=312&fod_list_no=2747 . Accessed 12 May 2014.

[5] Levinsky NG, Yu W, Ash A, Moskowitz M, Gazelle G, Saynina O, Emanuel EJ (2001). Influence of age on Medicare expenditures and medical care in the last year of life. JAMA.

[6] Payne G, Laporte A, Deber R, Coyte PC (2007). Counting backward to health care's future: using time-to-death modeling to identify changes in end-of-life morbidity and the impact of aging on health care expenditures. Milbank Q.

[7] Polder JJ, Barendregt JJ, van Oers H (2006). Health care costs in the last year of life--the Dutch experience. Soc Sci Med.

[8] Tang ST, Liu TW, Shyu YI, Huang EW, Koong SL, Hsiao SC (2012). Impact of age on end-of-life care for adult Taiwanese cancer decedents, 2001-2006. Palliat Med.

[9] Earle CC, Neville BA, Landrum MB, Ayanian JZ, Block SD, Weeks JC (2004). Trends in the aggressiveness of cancer care near the end of life. J Clin Oncol.

[10] Barbera L, Paszat L, Qiu F (2008). End-of-life care in lung cancer patients in Ontario: aggressiveness of care in the population and a description of hospital admissions. J Pain Symptom Manag.

[11] Emanuel EJ, Young-Xu Y, Levinsky NG, Gazelle G, Saynina O, Ash AS (2003). Chemotherapy use among Medicare beneficiaries at the end of life. Ann Intern Med.

[12] Dy SM, Wolff JL, Frick KD (2007). Patient characteristics and end-of-life health care utilization among Medicare beneficiaries in 1989 and 1999. Med Care.

[13] Sharma G, Freeman J, Zhang D, Goodwin JS (2008). Trends in end-of-life ICU use among older adults with advanced lung cancer. Chest.

[14] Van den Block L, Deschepper R, Drieskens K, Bauwens S, Bilsen J, Bossuyt N, Deliens L (2007). Hospitalisations at the end of life: using a sentinel surveillance network to study hospital use and associated patient, disease and healthcare factors. BMC Health Serv Res.

[15] Goodlin SJ, Zhong Z, Lynn J, Fago JP, Desbiens N, Connors AF, Connors AF, Wenger NS, Phillips RS (1999). Factors associated with use of cardiopulmonary resuscitation in seriously ill hospitalized adults. JAMA.

[16] Chalfin DB, Carlon GC (1990). Age and utilization of intensive care unit resources of critically ill cancer patients. Crit Care Med.

[17] Hamel MB, Davis RB, Teno JM, Knaus WA, Lynn J, Harrell F, Galanos AN, Wu AW, Phillips RS (1999). Older age, aggressiveness of care, and survival for seriously ill, hospitalized adults. SUPPORT investigators. Study to understand prognoses and preferences for outcomes and risks of treatments. Ann Intern Med.

[18] Moorin RE, Holman CD (2008). The cost of in-patient care in Western Australia in the last years of life: a population-based data linkage study. Health Policy.

[19] Masuda Y, Noguchi H, Kuzuya M, Inoue A, Hirakawa Y, Iguchi A, Uemura K (2006). Comparison of medical treatments for the dying in a hospice and a geriatric hospital in Japan. J Palliat Med.

[20] Earle CC, Park ER, Lai B, Weeks JC, Ayanian JZ, Block S (2003). Identifying potential indicators of the quality of end-of-life cancer care from administrative data. J Clin Oncol.

[21] Earle CC, Neville BA, Landrum MB, Souza JM, Weeks JC, Block SD, Grunfeld E, Ayanian JZ (2005). Evaluating claims-based indicators of the intensity of end-of-life cancer care. Int J Qual Health Care.

[22] Grunfeld E, Lethbridge L, Dewar R, Lawson B, Paszat LF, Johnston G, Burge F, McIntyre P, Earle CC (2006). Towards using administrative databases to measure population-based indicators of quality of end-of-life care: testing the methodology. Palliat Med.

[23] Grunfeld E, Urquhart R, Mykhalovskiy E, Folkes A, Johnston G, Burge FI, Earle CC, Dent S (2008). Toward population-based indicators of quality end-of-life care: testing stakeholder agreement. Cancer.

[24] Temel JS, Greer JA, Muzikansky A, Gallagher ER, Admane S, Jackson VA, Dahlin CM, Blinderman CD, Jacobsen J, Pirl WF (2010). Early palliative care for patients with metastatic non-small-cell lung cancer. N Engl J Med.

[25] Bakitas M, Lyons KD, Hegel MT, Balan S, Brokaw FC, Seville J, Hull JG, Li Z, Tosteson TD, Byock IR (2009). Effects of a palliative care intervention on clinical outcomes in patients with advanced cancer: the project ENABLE II randomized controlled trial. JAMA.

[26] Elkin EB, Bach PB (2010). Cancer's next frontier: addressing high and increasing costs. JAMA.

[27] Kung CM, Mo LR, Yan YH (2012). Consumption of national health insurance medical resources by hepatocellular carcinoma patients treated using radiofrequency ablation therapy. Asia Pac J Clin Oncol.

[28] Langton JM, Blanch B, Drew AK, Haas M, Ingham JM, Pearson SA (2014). Retrospective studies of end-of-life resource utilization and costs in cancer care using health administrative data: a systematic review. Palliat Med.

[29] Yabroff KR, Lamont EB, Mariotto A, Warren JL, Topor M, Meekins A, Brown ML (2008). Cost of care for elderly cancer patients in the United States. J Natl Cancer Inst.

[30] Riley GF, Lubitz JD (2010). Long-term trends in Medicare payments in the last year of life. Health Serv Res.

[31] Chastek B, Harley C, Kallich J, Newcomer L, Paoli CJ, Teitelbaum AH (2012). Health care costs for patients with cancer at the end of life. J Oncol Pract.

[32] McCall N (1984). Utilization and costs of Medicare services by beneficiaries in their last year of life. Med Care.

[33] Department of Statistics, Ministry of the Interior’s. http://www.moi.gov.tw/stat/news_content.aspx?sn=8664. Accessed 16 Dec 2015.

[34] National Health Insurance Research Database (NHIRD) Taiwan. http://nhird.nhri.org.tw/date_01.html. Accessed 22 May 2014.

[35] Lin CC, Lai MS, Syu CY, Chang SC, Tseng FY (2005). Accuracy of diabetes diagnosis in health insurance claims data in Taiwan. J Formos Med Assoc.

[36] Yu KH, Kuo CF, Luo SF, See LC, Chou IJ, Chang HC, Chiou MJ (2012). Risk of end-stage renal disease associated with gout: a nationwide population study. Arthritis Res Ther.

[37] Su TC, Bai CH, Chang HY, You SL, Chien KL, Chen MF, Chen HJ, Pan WH, Tseng CH, Cheng SH (2008). Evidence for improved control of hypertension in Taiwan: 1993-2002. J Hypertens.

[38] Charlson ME, Pompei P, Ales KL, MacKenzie CR (1987). A new method of classifying prognostic comorbidity in longitudinal studies: development and validation. J Chronic Dis.

[39] Deyo RA, Cherkin DC, Ciol MA (1992). Adapting a clinical comorbidity index for use with ICD-9-CM administrative databases. J Clin Epidemiol.

[40] Klabunde CN, Potosky AL, Legler JM, Warren JL (2000). Development of a comorbidity index using physician claims data. J Clin Epidemiol.

[41] Yee TW (2004). Quantile regression via vector generalized additive models. Stat Med.

[42] Carlson MD, Herrin J, Du Q, Epstein AJ, Barry CL, Morrison RS, Back AL, Bradley EH (2010). Impact of hospice disenrollment on health care use and medicare expenditures for patients with cancer. J Clin Oncol.

[43] Zhang B, Wright AA, Huskamp HA, Nilsson ME, Maciejewski ML, Earle CC, Block SD, Maciejewski PK, Prigerson HG (2009). Health care costs in the last week of life: associations with end-of-life conversations. Arch Intern Med.

[44] Singal BM, Hedges JR, Rousseau EW, Sanders AB, Berstein E, McNamara RM, Hogan TM (1992). Geriatric patient emergency visits. Part I: comparison of visits by geriatric and younger patients. Ann Emerg Med.

[45] Tsukioka G, Kakizaki S, Sohara N, Sato K, Takagi H, Arai H, Abe T, Toyoda M, Katakai K, Kojima A (2006). Hepatocellular carcinoma in extremely elderly patients: an analysis of clinical characteristics, prognosis and patient survival. World J Gastroenterol.

[46] Harrington SE, Smith TJ (2008). The role of chemotherapy at the end of life: “when is enough, enough?”. JAMA.

[47] Smith TJ, Hillner BE (2011). Bending the cost curve in cancer care. N Engl J Med.

[48] Hu W, Yasui Y, White J, Winget M (2014). Aggressiveness of end-of-life care for patients with colorectal cancer in Alberta, Canada: 2006-2009. J Pain Symptom Manag.

[49] Legler A, Bradley EH, Carlson MD (2011). The effect of comorbidity burden on health care utilization for patients with cancer using hospice. J Palliat Med.

[50] Burge FI, Lawson BJ, Johnston GM, Grunfeld E (2008). A population-based study of age inequalities in access to palliative care among cancer patients. Med Care.

