# Supplementary material for: Predictors of high healthcare costs in elderly patients with liver cancer in end-of-life: a longitudinal population-based study
Source: BMC Cancer. 2017 Aug 24;17:568. doi: 10.1186/s12885-017-3561-5 (PMC5571574; doi:10.1186/s12885-017-3561-5)
Supplement: Additional file 1: Table S1. — The codes to calculate the probability of high healthcare cost (HCs) for elderly patients with liver cancer based on our multiple logistic regression model. (DOC 30 kb) [file 12885_2017_3561_MOESM1_ESM.doc]

**Additional file 1:**

**Table S1: The codes to calculate the probability of high healthcare cost (HCs) for elderly patients with liver cancer based on our multiple logistic regression model.**

OpenOfficeCalc, Microsoft Excel, and R environments can be used to calculate the probability of high healthcare cost (HCs) for elderly patients with liver cancer based on our multiple logistic regression model.

1. In OpenOfficeCalc or Microsoft Excel:

Key in the values for ICU (yes = 1, no = 0) in the A1 cell, ventilator (yes = 1, no = 0) in the A2 cell, anticancer treatments (yes = 1, no = 0) in the A3 cell, admission days in the A4 cell, admission times in the A5 cell, CKD (yes = 1, no = 0) in the A6 cell, EVB (yes = 1, no = 0) in the A7 cell, opioid use (yes = 1, no = 0) in the A8 cell, ascites (yes = 1, no = 0) in the A9 cell, and hypertension (yes = 1, no = 0) in the A10 cell. Key in the following formula in any empty cell on the same spreadsheet to obtain the estimated probability of HC:

  = 1/ (EXP (-(-6.92 + 2.59*A1 + 1.24*A2 + 1.00*A3 + 0.22*A4 + (-0.85)*A5 + 1.07*A6 + 0.81*A7 + 0.61*A8 + (-1.07)*A9 + (-0.52)*A10 )) +1)

1. In an R environment:

To calculate the probability of HCs for elderly patients with liver cancer, substitute the values for the variables X1 to X10 in the following regression equation and execute in the R console:

yhat<- (-6.92 # constant

+ 2.59*X1       #X1= ICU, (yes = 1, no = 0)

+ 1.24*X2     # X2 = ventilator, (yes = 1, no = 0)

+ 1.00*X3      # X3 = anti-cancer treatments, (yes = 1, no = 0)

+ 0.22*X4       # X4 = admission days

+ (-0.85)*X5    # X5 = admission times

+ 1.07*X6       # X6 = CKD, (yes = 1, no = 0)

+ 0.81*X7       # X7 = EVB, (yes = 1, no = 0)

+ 0.61*X8       # X8 = opioids use, (yes = 1, no = 0)

+ (-1.08)*X10 #X9 = ascites, (yes = 1, no = 0)

+ (-1.52)* X11 # X10 = hypertension,(yes = 1, no = 0)

)

phat <- 1/(1 + exp(-(yhat)))

phat
